# Pandemic-Related Impairment in the Monitoring of Patients With Hypertension and Diabetes and the Development of a Digital Solution for the Community Health Worker: Quasiexperimental and Implementation Study

**DOI:** 10.2196/35216

**Published:** 2022-03-29

**Authors:** Christiane Correa Rodrigues Cimini, Junia Xavier Maia, Magda Carvalho Pires, Leonardo Bonisson Ribeiro, Vânia Soares de Oliveira e Almeida Pinto, James Batchelor, Antonio Luiz Pinho Ribeiro, Milena Soriano Marcolino

**Affiliations:** 1 Medical School and Telehealth Center Universidade Federal dos Vales do Jequitinhonha e Mucuri Teófilo Otoni-MG Brazil; 2 Telehealth Center Hospital das Clínicas Faculdade de Medicina, Universidade Federal de Minas Gerais Belo Horizonte Brazil; 3 Department of Statistics Universidade Federal de Minas Gerais Belo Horizonte Brazil; 4 Clinical Informatics Research Unit Faculty of Medicine University of Southampton Southampton United Kingdom; 5 Telehealth Center and Cardiology Service Hospital das Clínicas Faculdade de Medicina, Universidade Federal de Minas Gerais Belo Horizonte Brazil

**Keywords:** hypertension, diabetes mellitus, COVID-19, pandemic, primary health care, telemedicine, clinical decision support systems, patient care management

## Abstract

**Background:**

The restrictions imposed by the COVID-19 pandemic reduced health service access by patients with chronic diseases. The discontinuity of care is a cause of great concern, mainly in vulnerable regions.

**Objective:**

This study aimed to assess the impact of the COVID-19 pandemic on people with hypertension and diabetes mellitus (DM) regarding the frequency of consultations and whether their disease was kept under control. The study also aimed to develop and implement a digital solution to improve monitoring at home.

**Methods:**

This is a multimethodological study. A quasiexperimental evaluation assessed the impact of the pandemic on the frequency of consultations and control of patients with hypertension and DM in 34 primary health care centers in 10 municipalities. Then, an implementation study developed an app with a decision support system (DSS) for community health workers (CHWs) to identify and address at-risk patients with uncontrolled hypertension or DM. An expert panel assessment evaluated feasibility, usability, and utility of the software.

**Results:**

Of 5070 patients, 4810 (94.87%) had hypertension, 1371 (27.04%) had DM, and 1111 (21.91%) had both diseases. There was a significant reduction in the weekly number of consultations (107, IQR 60.0-153.0 before vs 20.0, IQR 7.0-29.0 after social restriction; *P*<.001). Only 15.23% (772/5070) of all patients returned for a consultation during the pandemic. Individuals with hypertension had lower systolic (120.0, IQR 120.0-140.0 mm Hg) and diastolic (80.0, IQR 80.0-80.0 mm Hg) blood pressure than those who did not return (130.0, IQR 120.0-140.0 mm Hg and 80.0, IQR 80.0-90.0 mm Hg, respectively; *P*<.001). Also, those who returned had a higher proportion of controlled hypertension (64.3% vs 52.8%). For DM, there were no differences in glycohemoglobin levels. Concerning the DSS, the experts agreed that the CHWs can easily incorporate it into their routines and the app can identify patients at risk and improve treatment.

**Conclusions:**

The COVID-19 pandemic caused a significant drop in the number of consultations for patients with hypertension and DM in primary care. A DSS for CHW has proved to be feasible, useful, and easily incorporated into their routines.

## Introduction

The COVID-19 pandemic severely hit health care systems worldwide, challenged their responsiveness, and forced them to redistribute human and material resources to emergency services and intensive care units dedicated to COVID-19 patients [1]. This emergency reorganization had a negative impact on monitoring people with noncommunicable chronic diseases (NCDs), such as hypertension and diabetes mellitus (DM), which need continuous follow-up [2].

Mortality from NCDs in low-income and medium-income countries is high due to the limitations of health systems in providing treatment for these diseases [3]. Hypertension is the main modifiable risk factor, with an independent association with cardiovascular disease, chronic kidney disease, and premature death [4] and risk factors for severe COVID-19 and COVID-19 mortality [5,6]. Social restriction intensified risky behaviors, including increased sedentary lifestyles, time in front of screens [7], ultraprocessed food consumption, and number of cigarettes smoked [8]. These habits contribute to weight gain as well as uncontrolled blood pressure (BP) and glucose levels of individuals with hypertension and DM, respectively [9].

This global impact of COVID-19 is especially favorable for the adoption of digital solutions in response to the challenges that the pandemic has imposed. They can be used not only to follow people with suspected or confirmed of COVID-19 but also to monitor patients with other diseases and provide essential health care services at the community level. Therefore, the pandemic has led to rapid development and utilization of mobile health (mHealth) apps [10,11], although these tools have been available for a long time.

Since June 2017, our group has been conducting a study that follows up people with hypertension and DM in Northeastern Minas Gerais, Brazil, a resource-constrained region called Vale do Mucuri (Mucuri Valley). Until October 2018, the HealthRise project, funded by the Medtronic Foundation, aimed to improve the screening and disease control of people with hypertension and DM [12]. The main activities were (1) training the multidisciplinary family health team, (2) organizing the flow of spontaneous and scheduled consultations, (3) expanding rational access to complementary exams, (4) supporting group activities, (5) sending text messages to patients’ cell phones, and (6) developing and implementing a clinical decision support system (CDSS). Nurses and physicians applied recommendations from evidence-based guidelines in their work routine providing the patient with up-to-date treatment. Community health workers (CHWs) received tablets to enroll patients in the screening phase. These devices were also useful to improve CHWs’ work routines, facilitating the entry of data into the Ministry of Health's information system [13].

There was a 2-month transition between the end of the HealthRise project and the beginning of the next project, the Charming Project (Control of Hypertension and Diabetes in Minas Gerais). This transition lasted until December 2018, and, in early 2019, the intervention restarted as the Charming Project, maintaining all the previous components and activities in the same territory.

In the pandemic scenario, which puts at risk the monitoring of those patients in the primary care setting, the purpose of this study was to assess the impact of the COVID-19 pandemic on the frequency of consultations for patients with hypertension and DM and the control of both diseases in a vulnerable region. Additionally, to mitigate the negative impact that the pandemic may have had on these patients, this study evaluated the implementation of a simple and efficient mHealth strategy for CHWs that was used during home visits, to prioritize the in-person consultation of patients with uncontrolled disease.

## Methods

### Study Design

This mixed methods study was a substudy of the Charming Project that took place in 34 primary health care centers (PHCCs) in 10 municipalities of Mucuri Valley: Ataléia, Catuji, Crisólita, Frei Gaspar, Itaipé, Ladainha, Novo Oriente de Minas, Ouro Verde de Minas, Setubinha, and Teófilo Otoni [13]. Mucuri Valley is part of the Northeast Macro-Region of Minas Gerais, Brazil, with a territorial extension of 24,781.5 km^2^ and a population of 516,073 inhabitants, marked by drastic socioeconomic contrasts, high rates of illiteracy and poverty, and low rates of control of hypertension and DM [14].

**Figure 1:**
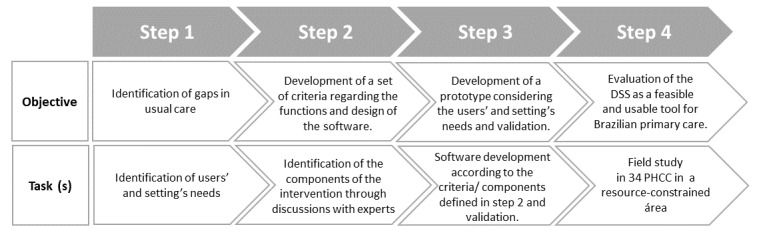
Flowchart of the study methodology. DSS: decision support system. PHCC: primary health care center.

This study was performed in 4 steps, according to the Medical Research Council framework (Figure 1): (1) identification of gaps in usual care, (2) identification of the components of the intervention through discussions with experts, (3) software development and validation, and (4) pilot testing [15].

### Step 1: Identification of Gaps in Usual Care

Local health authorities confirmed the first case of COVID-19 in Mucuri Valley on April 22, 2020. However, as soon as the World Health Organization (WHO) declared the COVID-19 a global pandemic on March 11, 2020, there was a significant drop in the number of consultations at the PHCC. At the beginning of the pandemic, the Ministry of Health’s recommendations were contradictory, and the role of CHWs was not established [16]. In May, general guidelines from the Ministry of Health suspended group activities and home visits, and PHCCs were designated to receive people with respiratory symptoms. Patients with chronic diseases were left in the background, receiving only prescription renewal. Consequently, there was a mischaracterizing of the work routine in the primary care setting.

### Quasiexperimental Study

The quasiexperimental study aimed to assess the impact of the COVID-19 pandemic on the frequency of consultations. Likewise, it also aimed to assess the impact of the pandemic on the control of those patients. Therefore, 2 periods were considered: the so-called period 1, from baseline (June 1, 2017) to March 13, 2020, and period 2, from March 14, 2020 (12th epidemiological week, when social restrictions were intensified) to December 31, 2020. This assessment included all patients followed by the HealthRise and Charming Projects with hypertension and DM aged 30 years to 69 years at the project's baseline [12], being monitored in the 34 PHCCs of 10 municipalities who had at least two consultations. The age range was previously determined by the funder and described in the public call for project submission.

Data were obtained through the usual medical and nursing consultation procedures, recorded in the software developed in the study [13]. Variables of interest were sociodemographic data (age, sex, education, income), clinical data (hypertension, DM, stroke, peripheral arterial disease, coronary artery disease, heart failure, alcoholism, physical inactivity, smoking), laboratory data (glycated hemoglobin [HbA_1c_]), physical examination measures (systolic BP [SBP] and diastolic BP [DBP]), and follow-up data (number of consultations performed). HbA_1c_ was assessed using laboratory tests of peripheral blood samples and point-of-care tests.

### Step 2: Identification of the Components of the Intervention

#### mHealth Solution for CHWs

Since primary health care professionals had already used digital solutions in the previous projects, an mHealth solution was planned for CHW that could identify patients with uncontrolled hypertension and DM at home. From the recognition of these patients, the CHW could prioritize them for medical consultation at the PHCC. The app was developed to run on tablets and smartphones and has a DSS that indicates to the CHW whether the patient’s disease is controlled. By entering simple data, the CHW receives immediate feedback during the home visit, providing prompt patient guidance.

The use of synchronous teleconsultations as part of this intervention was considered. However, we detected many barriers that impaired their implementation, such as a significant proportion of illiteracy among the population, social and economic vulnerability, poor internet connectivity in remote areas, and lack of infrastructure.

#### Procedures

To assess patients with hypertension, the CHW used an automatic arm BP monitor (OMRON HEM-7320). However, in Brazil, CHWs’ basic training for their role does not include performing nursing procedures, such as measuring capillary blood glucose [17]. So, in cases of DM, they guided patients to use dipsticks to assess glycosuria in an isolated urine sample. It made screening the most uncontrolled cases (urine glucose concentrations of ++ or more corresponds to blood glucose above 250 mg/dL) possible. In addition, some patients with uncontrolled DM received test strips and instructions to perform glucose self-monitoring and improve the adjustments to the insulin prescription. Using tablets, CHW entered all the information obtained into the app, which consists of a simple questionnaire and a DSS. When the DSS classifies the patient as uncontrolled, a message advises the CHW to make a medical appointment at the PHCC.

Evaluation of glycemic control by HbA_1c_, using a point-of-care portable HbA_1c_ analyzer, was available in many PHCCs for testing just before the medical consultation, with immediate results, and allowing prompt decision-making.

#### Prioritization Criteria

Since it would not be possible to attend to all patients with hypertension and DM, prioritization criteria were established according to the findings expected in home visits. The following patients were prioritized for in-person medical consultations at the PHCC: people with hypertension and SBP ≥160 mm Hg or DBP ≥100 mm Hg and/or people with DM and any capillary blood glucose measurement ≥250 mg/dL or glycosuria result ++ or higher.

The intervention was centralized in the CHW because of their close contact with the community through home visits, activities in support groups, and performance of preventive actions. They know about the families’ health and social conditions, adherence to treatment, and attendance at consultations at the PHCC [17,18].

### Step 3: Software Development and Validation

#### App Overview

The purpose of the app was to identify, in the home environment, patients who were seriously decompensated or at higher risk of decompensation and to prioritize them for medical consultation at the PHCCs.

To ensure privacy, data are kept encrypted on the tablet. User sessions expire whenever devices enter the sleep mode or periodically (the shortest of times). User digital authentication is secure. The app has features to operate in online and offline modes. Due to the lack of internet connection in patients’ homes where data are collected and lack of 3G or 4G connection in the tablets, it is necessary to store patient data used to promptly generate decision support in the device. This information is uploaded online and as soon as CHWs go back to the PHCC.

To develop the app, the developer team and stakeholders had a round of meetings in order to define its scope. Bearing in mind the prospective user profile, Android 4.4 or above (Api level 19, KitKat) was selected as the operating system. A prototype was made and submitted for approval, upon which the development phase began. Netbeans and Java for Android were used together with the libraries Firebase Crashlytics, Analytics, Volley Plus, and Realm Database, the latter for the local mobile databank. REST was used for mobile-databank (POSTGRESQL) communication. The development phase was incremental, and each successive version was submitted to stakeholders for testing and approval. The last submission included a test battery with final users, the results of which were reported to the developer team for error solution. Once a final submission was approved, the app was released for production. The app can be downloaded from Google Play Store. Support for installation and use was provided by the local team.

The app is freely available in Google Play Store and can be downloaded under the name *Questionário Charming*. At the moment, only the Portuguese version is available.

#### App Content and Functionality

The app consists of (1) a log-in screen, (2) a patient search screen, (3) a patient registration screen, (4) patient assessment, and (5) decision support.

**Figure 2:**
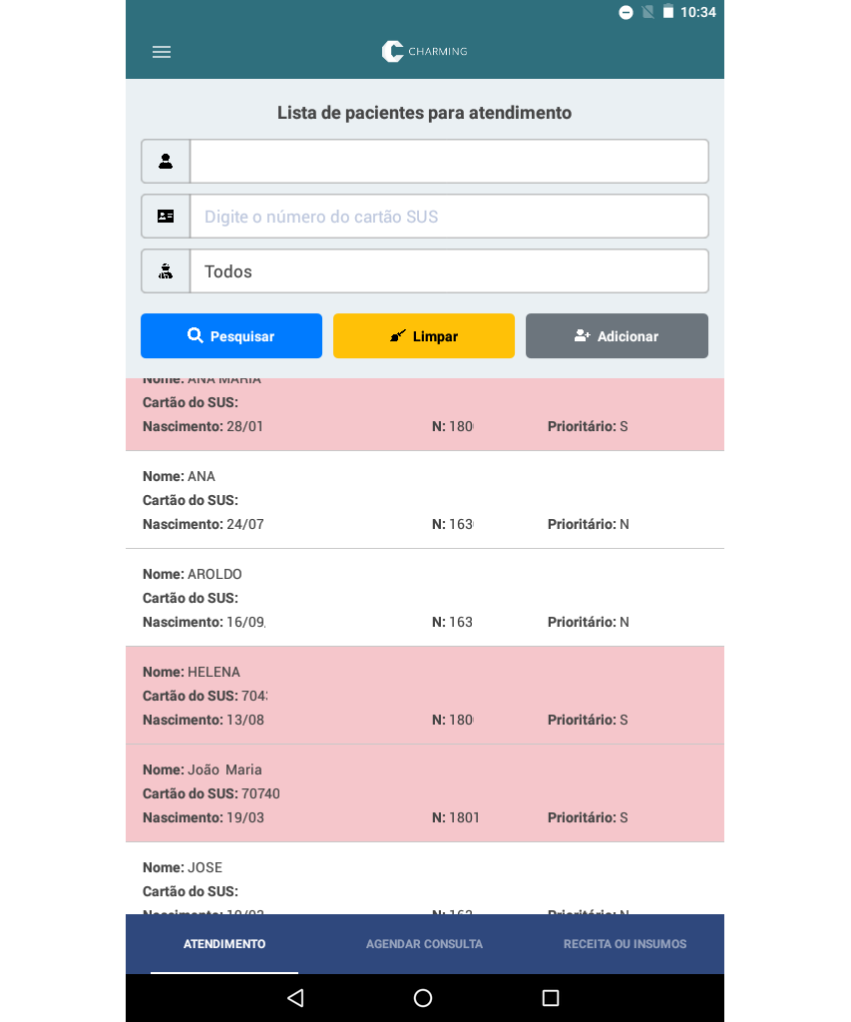
Example patient search screen with fictitious information: patient’s name, registration number in the public health system, birth date, record id, and priority, according to criteria explained in the text.

The login screen allows individualized access to the system through the credentials (user and password) provided to each professional. Once logged in, the professional has access to the screen to search for registered patients (Figure 2).

If the patient was not registered before, the CHW can create a new registration and input demographic data, address, telephone number, and information on diagnosis of hypertension and DM.

**Figure 3:**
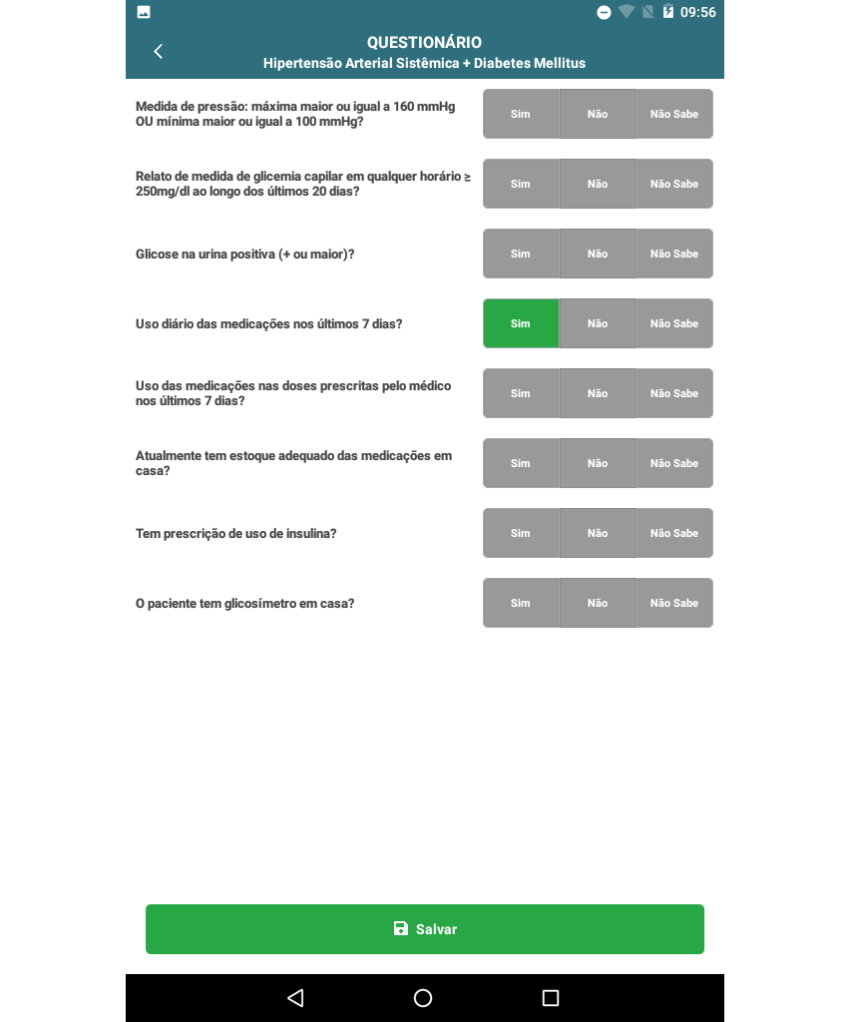
Patient assessment screen, on which the community health worker easily inputs information obtained during the home visit: blood pressure levels, glycosuria, medication adherence and access, insulin use, and presence of a glucometer at home.

After choosing a specific patient, the professional enters the questionnaire screen, which includes BP levels, recent capillary blood glucose levels, glycosuria result, questions about adherence to drug treatment, reasons for nonadherence (if it occurs), if the patient has an insulin prescription, and glucose strip supplies (Figure 3). It is worth mentioning that the Brazilian public health system (Sistema Único de Saúde [SUS]) provides glucometer and blood glucose strips for people with DM who use insulin.

**Figure 4:**
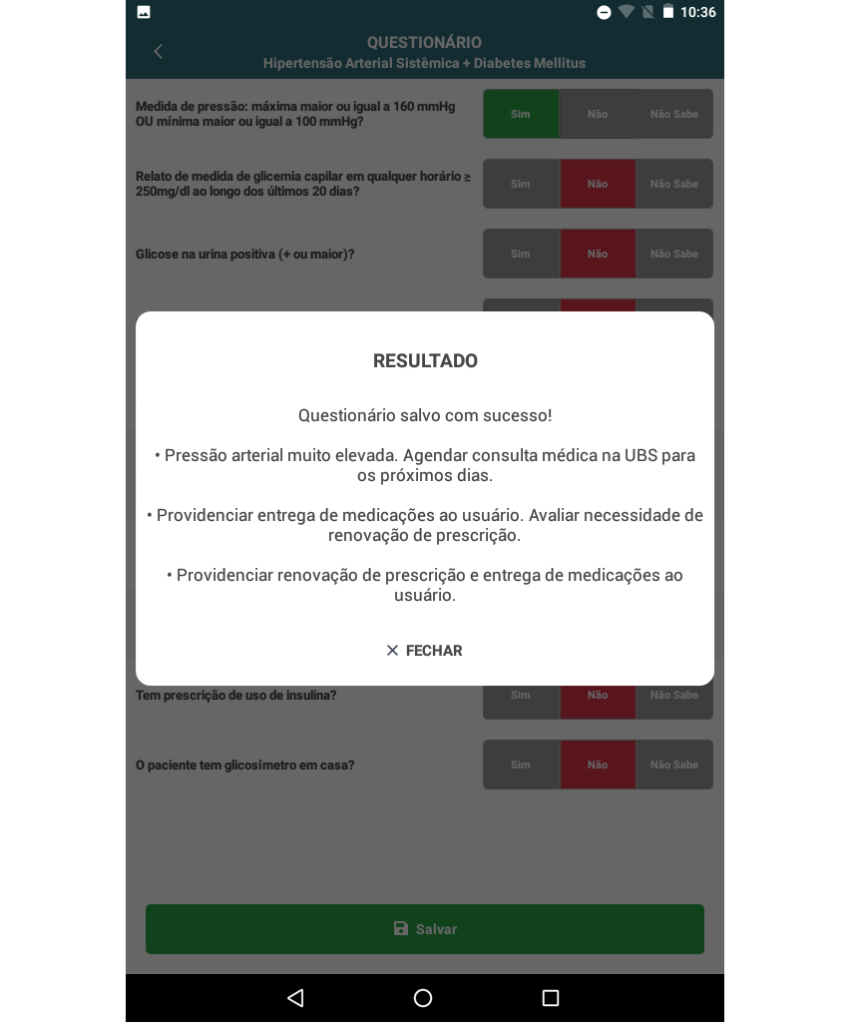
Decision support screen, which shows the best recommendation for the patient after the community health worker inputs and saves the patient data.

The DSS provides personalized recommendations, generated according to the data entered in the CHW evaluation. The messages alert the CHW if glucose or BP levels are high and suggests scheduling medical or nursing visits in the PHCCs, delivery of drugs or supplies, and prescription renewal (Figure 4).

#### Pretesting

In order to ensure that the system was operating as intended, with no bugs, and that the recommendation results matched the prespecified decision tree, the prototype was tested multiple times through manual insertion of test cases. Medical students, professors, and researchers took the tests several times.

#### Expert Panel Assessment

The expert panel consisted of 4 primary care physicians, 1 nurse, 1 pharmacist, and 1 CHW, all working in primary health care and 2 of them (1 of the physicians and the nurse) also working as health care managers. They were all recognized as technical references and completely independent of the researchers and implementation sites. The specialists tested the app for 1 week by simulating the most different situations that the CHW might encounter during a home visit. At the end of the tests, they gave their opinion through a questionnaire previously developed by our group [19]. The first part of the questionnaire included the following participant characteristics: sex at birth, age, education level, time since graduation, profession, role in primary care, prior knowledge of information technology (IT), ease of use, and frequency of internet use, for how long during the day, and for what reasons (personal, professional, or other). The second part included Likert scale questions, varying from 1 (strongly disagree) to 5 (strongly agree), to assess feasibility, usability, and utility. 

### Step 4: Pilot Testing

The IT team and researchers carried out the implementation, through remote and in-person training. CHWs in the 10 municipalities received specific remote training about the (1) correct use of personal protective equipment, (2) identification of suspected cases of COVID-19 during home visits, (3) BP measurement using an automatic arm BP monitor, (4) glycosuria assessment in an isolated urine sample using reagent dipsticks, and (5) identification of cases with uncontrolled hypertension and DM during home visits. Depending on the clinical situation, they had to measure BP and/or glycosuria and guide patients with DM about how to measure capillary blood glucose at home. They underwent a specific training session through a web conference to clarify the expansion of restrictive measures regarding the COVID-19 pandemic. In addition, CHWs received supporting material about the software and instructions for use. The IT team installed the app on the tablets that CHWs were already using. During these visits to the PHCCs, they provided hands-on training on how to use the app and answered CHWs’ remaining doubts. Later when devices, app incompatibilities, and login-related issues arose, the IT team promptly solved them remotely.

### Statistical Analysis

Continuous variables were described by measures of central tendency (mean or median), dispersion (SD or IQR), and amplitude, according to the distribution assessed using the Kolmogorov-Sminov test. Categorical variables were described with measures of absolute and relative frequencies. Using the Chow test, weekly consultations’ time series data were analyzed to identify possible structural changes (ie, sudden changes in the trend of the time series) related to the pandemic. Variables were compared between periods 1 and 2 using Student *t* tests, Mann-Whitney *U* tests, or Fisher exact tests, according to the normality of the distribution and type of variable. Statistical analysis was performed with R software (version 4.0.2) with the strucchange and ggplot2 packages.

### Ethical Review

The Federal University of Jequitinhonha and Mucuri Valleys’ Research Ethics Committee gave ethical approval for the study, which is registered under the Certificate of Presentation of Ethical Appreciation (CAAE) number 40479820.2.0000.5108. 

## Results

### Quasiexperimental Study

During the 183-week follow-up period ranging from June 2017 to December 2020, 17,345 consultations were carried out, with a median number of 2 consultations per patient, except for those with diagnoses of both hypertension and DM, who had a median of 3 consultations. Physicians (10,199/17,345, 58.8%) performed most of them. HbA_1c_ was evaluated through laboratory tests from peripheral blood samples and point-of-care tests. From the 3488 HbA_1c_ assessments, 1027 (29.4%) used point-of-care tests. Between 2019 and 2020, there was a 74% reduction in the number of HbA_1c_ tests (977 vs 255) and a 62.5% reduction in the number of BP measurements (3898 vs 1461). The overall number of patients was 5202. There were 4936 (94.9%) with hypertension and 1403 (27.0%) with DM. It is noteworthy that DM and hypertension coexisted in 1137 patients (Table 1).

The beginning of the 12th epidemiological week, on March 14, 2020, is a milestone for this study, as it corresponds with the moment of escalation of social restriction measures. The Chow test confirmed the break point, which divided the timeline into period 1 and period 2.

For the before-after assessment, 5070 patients were analyzed. Of these, 4810 (94.9%) patients had hypertension, and 1371 (27.0%) had DM. Among them, 1111 (23.1%) patients had both diseases. Most patients were female (3369/5070, 66.4%), with median age of 56.0 (IQR 48.0-62.0) years and median BMI of 27.9 (IQR 24.6-31.6) kg/m^2^ (Table 2).

It is noteworthy that DM and hypertension coexisted in 1111 patients.

There was a significant reduction in the number of consultations, BP measurements, and HbA_1c_ dosage between period 1 and period 2 (Table 3).

**Table 1:** Consultations, procedures, and patient data.

Characteristic		Overall sample (n=17,345), n (%)	By year, n			
			2017	2018	2019	2020
Number of consultations		17,345 (100)	1990	6325	6437	2593
**Number of consultations by health care professional**						
	Physician	10,201 (58.8)	1300	4393	3450	1058
	Nurse	7144 (41.2)	690	1932	2987	1535
Total number of procedures, n (%)		14,584 (84.1)	589	730	1389	780
**Procedures, n (%)**						
	HbA_1c_^a^ tests	2461 (16.9)	583	646	977	255
	HbA_1c_ POC^b^ tests	1027 (7.0)	6	84	412	525
	Blood pressure measurements	11,096 (76.1)	1580	4157	3898	1461
Total number of patients, n (%)		5202 (30.0)	1757	3444	2833	1576
**Number of patients with each disease, n (%)**						
	DM^c^	1403 (27.0)	491	911	913	599
	Hypertension	4936 (94.9)	1654	3288	2674	1479

^a^HbA_1c_: glycated hemoglobin.

^b^POC: point-of-care.

^c^DM: diabetes.

**Table 2:** Characteristics at baseline.

Characteristics		Overall (n=5070)			DM^a^ (n=1371)				Hypertension (n=4810)	
Gender (female), n (%)		3369 (66.4)			953 (69.5)				3203 (66.6)	
Age (years), median (IQR)		56.0 (48.0-62.0)			57.0 (49.0-63.0)				56.0 (48.0-63.0)	
BMI (kg/m^2^), median (IQR)		27.9 (24.6-31.6)^b^			28.5 (25.1-32.4)^c^				27.9 (24.6-31.6)^d^	
Number of consultations, median (IQR)		2.0 (1.0-5.0)			3.0 (2.0-6.0)				2.0 (1.0-5.0)	
**HBA_1c^e^_ tests**										
	Number of HbA_1c_ tests, median (IQR)	-^f^	-^f^	1.0 (1.0-3.0)				N/A^g^		N/A
	HbA_1c_ result (%), median (IQR)	-	-^f^	7.6 (6.4-9.6)^h^				N/A		N/A
	Number of HbA_1c_ results <7%, n (%)	-^f^	-^f^	409 (37.9)^h^				N/A		N/A
**BP^i^ measures**										
	Number of BP measures, median (IQR)	-^j^	-^j^	N/A		N/A	1.0 (1.0-3.0)			
	SBP^k^ (mm Hg), median (IQR)	-^j^	-^j^	N/A		N/A	130.0 (120.0-140.0)^l^			
	DBP^m^ (mm Hg), median (IQR)	-^j^	-^j^	N/A		N/A	80.0 (80.0-90.0)^l^			
	SBP <140 mm Hg and DBP <90 mm Hg, n (%)	-^j^	-^j^	N/A		N/A	1937 (48.7)^l^			

^a^DM: diabetes mellitus.

^b^n=3961 (78.1%).

^c^n=1074 (78.3%).

^d^n=3759 (78.2%).

^e^HbA_1c_: glycated hemoglobin.

^f^Only tested in those with DM, so the values would be the same as those reported under DM.

^g^N/A: not applicable.

^h^n=1079 (78.7%).

^i^BP: blood pressure.

^j^Only tested in those with hypertension, so the values would be the same as those reported under hypertension.

^k^SBP: systolic blood pressure.

^l^n=3980 (82.7%).

^m^DBP: diastolic blood pressure.

**Table 3:** Weekly number of consultations, blood pressure measurements, and glycated hemoglobin (HbA1c) tests in period 1 and period 2.

Characteristic	Overall (n=183), median (IQR)	Period 1 (n=142) , median (IQR)	Period 2 (n=41) , median (IQR)	*P* value
Consultations	83.0 (29.0-139.5)	107.0 (60.0-153.0)	20.0 (7.0-29.0)	<.001
BP^a^ measures	56.0 (22.5-89.5)	68.0 (38.2-99.0)	11.0 (4.0-19.0)	<.001
HbA_1c_ tests	13.0 (3.5-29.0)	16.5 (5.0-32.8)	4.0 (0.0-22.0)	<.001

^a^BP: blood pressure.

**Figure 5:**
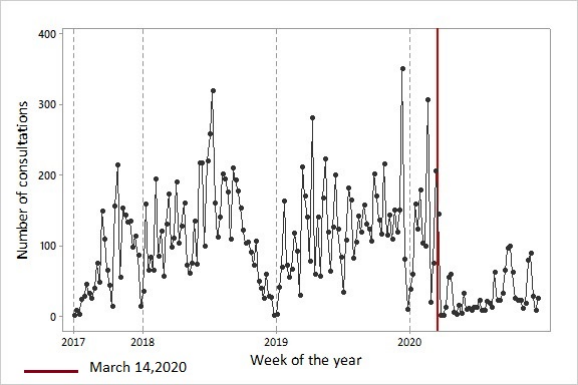
Weekly number of consultations and variations between 2017 and 2020.

Over the 183 weeks of follow-up in the series, there were other variations in the number of consultations in specific periods. In 2017, with the beginning of patient follow-up, there was a progressive growth in the number of weekly appointments at PHCCs. At the end of the year, there was an abrupt drop in the number of consultations, which is systematically observed in all years. In 2018, the number of consultations tended to remain stable, but there was a sudden drop in October, which corresponded with the transition between the HealthRise and Charming projects, followed by the expected reduction in attendance at the end of the year. In early 2019, when funding was reinstated, the number of consultations rose again. In 2019, the pattern of consultations presented the usual variations, and in the first 2 months of 2020, activities in the PHCCs were normal. However, at the beginning of the 12th epidemiological week, there was a dramatic reduction in the number of consultations at PHCCs. On April 22, 2020, the first COVID-19 case in the region was confirmed. Throughout 2020, a reduced number of weekly consultations is clearly observed (Figure 5).

Of the 5070 patients who were being followed since before the pandemic, 4298 (84.8%) did not return for a consultation after the social distancing measures were implemented. Of the 772 (15.2%) individuals who returned, the median time between the last consultation before the social distancing measures and the first consultation after it was 233 days (Table 4).

It is noteworthy that DM and hypertension coexisted in 313 patients who returned for a consultation in period 2.

The proportion of patients who returned after period 2 was much lower than the proportion observed during period 1 (772/5070, 15.2% vs 2565/5070, 50.6%; Table 5).

It is noteworthy that DM and hypertension coexisted in 1111 patients.

The characteristics common to the groups of those who did not return for consultation in period 2 and those who did return were compared. The median age of those who returned was slightly higher—58.0 (IQR 51.0-65.0) years versus 56.0 (IQR 48.0-63.0) years (*P*<.001)—but there was no difference regarding their sex. The median number of consultations in period 1 was also higher in that population: median 5.0 (IQR 3.0-7.0) vs 2.0 (IQR 1.0-4.0). Lower median SBP levels—120.0 (IQR 120.0-140.0) mm Hg versus 130.0 (IQR 120.0-140.0) mm Hg—and median DBP levels—80.0 (IQR 80.0-80.0) mm Hg versus 80.0 (IQR 80.0-90.0) mm Hg—were found in patients who returned for consultation, as well as a higher proportion of patients with controlled hypertension (431/772 64.3% vs 1735/4298, 52.8%). However, among people with DM, there was no difference in HbA_1c_ levels between periods 1 and 2 (Table 6).

It is noteworthy that DM and hypertension coexisted in 798 patients who did not return and 313 patients who returned.

Since June 2017, 110 patients were discharged from the system: 35 due to formal withdrawal, 62 who moved, 12 who died from a nontraumatic cause, and 1 from a traumatic cause.

**Table 4:** Time between the last consultation in period 1 and the first consultation in period 2.

Measurement	Overall (n=772)	DM^a^ (n=366)	Hypertension (n=719)
Time (days), median (IQR)	233.0 (122.8-356.0)	206.0 (114.0-306.5)	238.0 (125.0-362.0)

^a^DM: diabetes mellitus.

**Table 5:** Comparison of returns for consultation at a primary health care center (PHCC) in periods 1 and 2.

Characteristic	Overall (n=5070)	DM^a^ (n=1371)	Hypertension (n=4810)
No return, n (%)	1732 (34.2)	297 (21.7)	1660 (34.5)
Return in period 1, n (%)	2566 (50.6)	708 (51.6)	2431 (50.5)
Return in period 2, n (%)	772 (15.2)	366 (26.7)	719 (14.9)

^a^DM: diabetes mellitus.

**Table 6:** Comparison of the characteristics of patients between those who returned and did not return in period 2.

Characteristic		Did not return (n=4298)	Returned (n=772)	*P* value
Gender, female n (%)		2849 (66.3)	520 (67.4)	.56
Age (years), median (IQR)		56.0 (48.0-63.0)	58.0 (51.0-65.0)	<.001
**Disease,** **n (%)**				
	Hypertension	4091 (95.2)	719 (93.1)	<.001
	DM^a^	1005 (23.4)	366 (47.4)	
BMI^b^, median (IQR)		27.9 (24.7-31.6)^c^	27.8 (24.6- 31.8)^d^	.88
Number of consultations before the pandemic, median (IQR)		2.0 (1.0-4.0)	5.0 (3.0-7.0)	<.001
**BP^e^ values**				
	SBP^f^ (mm Hg)^b^, median (IQR)	130.0 (120.0-140.0)^g^	120.0 (120.0-140.0)^h^	<.001
	DBP^i^ (mm Hg)^b^, median (IQR)	80.0 (80.0-90.0)^g^	80.0 (80.0-80.0)^h^	<.001
	SBP <140 mm Hg and DBP <90 mm Hg, n (%)^b^	1735 (52.8)^g^	431 (64.3)^h^	<.001
**HbA_1c_^j^**				
	HbA_1c_ result (%), median (IQR)^b^	7.6 (6.3-9.5)^k^	7.6 (6.5-9.3)^l^	.80
	HbA_1c_ <7%, n (%)^b^	272 (37.3) ^k^	108 (33.8)^l^	.28

^a^DM: diabetes mellitus.

^b^Last measure or test before the pandemic.

^c^n=3243 (75.2%).

^d^n=685 (88.7%).

^e^BP: blood pressure.

^f^SBP: systolic blood pressure.

^g^n=3286 (76.5%).

^h^n=670 (86.8%).

^i^DBP: diastolic blood pressure.

^j^HbA_1c_: glycated hemoglobin.

^k^n=730 (73%).

^l^n=320 (87%).

### Validation: Expert Panel Assessment

All 7 experts who participated fully agreed that the app could be used in primary care settings to improve care for people with hypertension and/or DM. They also agreed that it could be easily incorporated in work routines (median 4.0, IQR 4.0-5.0). All believed that the app does not cause significant delays in the daily routine. As for usability, the overall evaluation was good, but all professionals claimed that the app was not intuitive and previous training is necessary. As for utility, they believed that the app might improve the treatment and care of people with hypertension and DM. The characteristics of the experts and the results of the feasibility assessment can be seen in Multimedia Appendix 1 and Multimedia Appendix 2.

### Pilot Testing

From November 2020 to May 2021, 211 CHWs from 10 municipalities received training to evaluate patients with hypertension and DM during in-home visits. The implementation was carried out progressively, and the first records were on paper. With hands-on training in May 2021, CHWs started using the app and entering data obtained from home visits. From May 2021 to December 2021, there were 1314 records from CHWs’ in-home visits using the app: 1266 (96.4%) were patients with hypertension, 245 (18.6%) were patients with DM, and 197 (15.0%) were patients with both diseases. They found 220 (220/1266, 17.38%) patients with high BP (≥160 mm Hg or ≥100 mm Hg); 34 (34/245, 13.9%) patients reported capillary blood glucose level at any time in the last 20 days ≥250 mg/dL. Regarding glycosuria, 40 (40/245, 16.3%) had a positive result (Multimedia Appendix 3).

## Discussion

### Main Findings

We observed a significant reduction in the weekly number of consultations, BP measurements, and HbA_1c_ tests between periods 1 and 2. There was a systematic reduction in PHCC visits in all the last months of the years, regardless of the pandemic, due to the December holidays and summer vacations. The proportion of patients who returned for a medical consultation at the PHCCs was also significantly lower during the pandemic. Of those who returned, it was observed that they were more assiduous in follow-up appointments before the pandemic when compared to the period after isolation measures were implemented. Lower SBP and DBP levels were observed in patients who returned for consultation during the pandemic, as well as a higher proportion of patients with controlled hypertension. However, among people with DM, there was no difference in HbA_1c_ levels between periods 1 and 2.

The reduction in the number of consultations was observed globally. According to a survey by the WHO, completed by 155 countries in May 2020, 53% of them had partially or completely disrupted services for hypertension treatment and 49% for DM and DM-related complications at that time [20]. Another survey that included 202 health care professionals from 47 countries observed that DM was the chronic disease most affected by the reduction in health care resources due to COVID-19 [21]. A worldwide survey submitted from 909 centers performing cardiac diagnostic procedures in 108 countries found a 42% drop in the number of procedures from March 2019 to March 2020 and a 64% drop from March 2019 to April 2020 [22].

In Germany, during the lockdown, there was a dramatic reduction in the number of consultations with general practitioners, independent of age, sex, and location (rural vs urban areas) [23]. The immediate need for social restriction forced health care systems to adopt telemedicine for ensuring baseline and, in some selected cases, advanced health care support [24]. As a result, the COVID-19 pandemic has strengthened the use of telemedicine as an indispensable resource to monitor the health conditions of people at home, including those with hypertension [25]. In Italy, the number of visits to general practitioners’ offices and tests dropped markedly. At the same time, the number of home app users, exchanging data between patients and doctors, significantly increased. It thus resulted in significant improvement of BP control [26]. Telemedicine with video consultations was a significantly effective tool in the management of people with hypertension, with high levels of patient satisfaction in the United States [27]. Telemedicine consultations contributed to maintaining care for 69% people with antineutrophil cytoplasmic antibody–associated vasculitis, both in the United States and the United Kingdom, allowing monitoring and identification of deterioration at home [28]. With regards to DM, the role of telehealth in the care of people with type 1 DM has expanded dramatically during the COVID-19 pandemic [29]. Successful experiences have been described in India and Australia, where patients who attended through teleconsultations had slightly better glycemic control than those in the pre-COVID-19 period [30,31]. In the United States, although DM-related outpatient visits and testing fell during the pandemic, there was no evidence of a negative association with glycemic control. Telemedicine may have prevented substantive disruptions in medication prescribing [32].

In Brazil, the Ministry of Health has taken steps to ensure care for people with respiratory syndrome throughout the SUS health network, reinforcing primary care as the preferred gateway [33]. However, at the same time, the heterogeneity of the organization of primary care in Brazil [34] as well as the restrictions imposed by the pandemic [35] compromise the continuous monitoring of patients with chronic diseases. CHWs were prioritized for health surveillance and administrative actions within the PHCCs and even issues that are not their responsibility, such as helping with vaccination campaigns [16]. The problem was even more serious in remote and resource-constrained areas, where patients need to travel long distances to receive health care and medication. As the management of COVID-19 cases has become a priority for most health units, nonemergency medical services or services not related to COVID-19 have been postponed indefinitely, predisposing patients with hypertension and DM to a high potential for increased risk of complications and a worse prognosis [35].

The consequences of these changes can already be seen in Brazil. In the 6 capital cities that had the highest number of deaths from COVID-19, there was also an increase in the number of deaths from cardiovascular diseases. These findings were probably a consequence of the poor health services infrastructure and reflects the increase in home deaths due to the impaired access to health services [36]. Cardiovascular diseases have a significant relationship with COVID-19, both as risk factors and prognostic indicators and as complications [37,38]. Nevertheless, despite this, the pandemic forced a reorganization of health systems that compromised the provision of adequate and timely health services [39,40] and negatively affected mortality [41].

Facing the reality of patients at home and without proper care, a strategy using mHealth for CHWs was planned and implemented to identify patients at risk. These patients were referred to the PHCCs for medical consultation. CHWs were chosen as the central point of this intervention because they represent the bridge between the health system and the users, especially the most vulnerable, enabling the capillarity of the SUS. They live in the same community for which they care and ensure that the family health strategy is taken to the communities. Their responsibilities include education and health promotion, keeping records of individuals and families, identifying those at risk, making regular home visits to monitor children's vaccinations or the well-being of chronic patients, scheduling consultations with a maternal health specialist, advising on the correct use of medications, and contributing to mosquito control campaigns [42]. 

Although digital health was in use long before the pandemic, the challenges of the current moment encouraged its escalation, which is happening quickly. The options for digital tools are vast, including video visits, email, mobile phone apps, chatbots, voice interface systems, smartwatches, oxygen monitors, and thermometers [11]. mHealth technology has been proven to enhance the management of patients with chronic diseases [43] and is associated with a reduction in BP and better medication adherence for people with hypertension [44]. Also, mHealth‐based interventions for patients with type 1 DM significantly decreased HbA_1c_, improved life satisfaction, and improved mental health [45]

The use of digital tools by CHWs has been successfully carried out in several countries, not only to fight the pandemic but also to minimize the impact of interruptions in patient follow-up [46]. However, to be effective, digital health solutions for CHWs need to be easy to use. Furthermore, there is a need to improve their skills, improve accessibility, and ensure continuity of care [47]. For this, challenges such as training on new mHealth solutions, weak technical support, and issues of internet connectivity must be overcome [10,48]. The app is an easy-to-use digital tool, with objective questions and yes/no/don’t know answers. Therefore, education level is not an obstacle to using it. Likewise, we guarantee training and support for the use of the app, solving doubts online in real time.

The expert panel assessment classified the app as feasible and useful. However, they agreed that its use requires training. They believe in the app's potential to contribute to the identification of patients at risk and the provision of medication by health managers. This information is important to obtain regardless of the pandemic. The federal government guarantees the donation of glucometers for people with DM who use insulin, but the supply of strips is erratic. The app can provide important feedback regarding municipal financing and logistics for purchasing medicines. The measurement of BP by the CHW using a digital monitor adds value to the home visit. The professionals and the patients have accepted this innovation without restrictions. 

The pilot test results reflect a short evaluation period as the intervention itself is ongoing. The findings showed a low percentage of patients with uncontrolled hypertension and DM when evaluated at home. Medication adherence as well as the supply of medications were satisfactory. The aim of measuring BP, glycosuria, and blood glucose levels was to prioritize in-person medical consultation for those at risk and not just to identify people with uncontrolled disease. The major limitation was that the PHCCs were designed to receive people with respiratory symptoms as a priority. Therefore, it was not possible to schedule a medical consultation for all patients with hypertension and DM outside the control goals. As the app was designed to be simple, the CHW does not enter the patient's BP but only marks if it is ≥160 x 100 mm Hg. Therefore, people outside the control target whose BP is <160 x 100 mm Hg will not be referred to a PHCC, which can underestimate the number of patients outside the control goals. The same is true for DM, as only those with a change equivalent to blood glucose ≥250 mg/dL will be identified and referred for consultation. Another aspect that needs to be considered is that patients who participated in the pilot test are the ones who most frequently attended the PHCC. The researchers did not interfere in the choice of patients for the pilot test, but the CHW preferred to visit those with greater adherence to treatment. This justifies the finding of a low proportion of people with BP and glucose levels high enough to warrant a medical consultation and justifies the adequate supplies of medication.

### Limitations

The cutoff BP, glycosuria, and blood glucose values to indicate the need for a medical consultation were established to prioritize those most in need, since the PHCC could not attend to all patients. Therefore, the results cannot be seen as just a definition of the proportion of patients with uncontrolled disease, as this underestimates the real number of patients with uncontrolled disease.

### Next Steps

The integration of the CDSS with the current electronic medical record of the Brazilian public health system software (e-SUS) is a challenge, and we are currently working to overcome this barrier. We are planning to expand the project to PHCCs in other municipalities, even with the reduction of the pandemic. Our team believes that this is a promising strategy that values and expands the CHWs’ skills, strengthens their relationship with the community, promotes the identification and adequate referral of patients with uncontrolled disease, and brings the users even closer to the SUS. We expect services to be fully resumed in the near future, when we will be able to review the cut-off levels of BP and glycosuria or glycemia to refer to the PHCC. 

### Conclusions

The COVID-19 pandemic caused significant impairment in the follow-up of patients with hypertension and DM in a resource-constrained region of Brazil, due to the reduction in the number of medical and nursing consultations, BP measures, and HbA_1c_ assessments after the initiation of social restriction measures. A DSS app has proven to be feasible and useful, and CHWs are using it to identify patients with uncontrolled hypertension and DM who are at home and at risk.

## Appendix

Multimedia Appendix 1 Characteristics of Experts who validated the app (N=7).

Multimedia Appendix 2 Feasibility, usability and utility assessment.

Multimedia Appendix 3 Characteristics of the study patients.

## Abbreviations

BP: blood pressure
CDSS: clinical decision support system
CHW: community health workers
DBP: diastolic blood pressure
DM: diabetes mellitus
DSS: decision support system
HbA1c: glycated hemoglobin
IT: information technology
mHealth: mobile health
NCD: noncommunicable chronic disease
PHCC: primary health care center
SBP: systolic blood pressure
SUS: Sistema Único de Saúde (Brazilian public health system)
WHO: World Health Organization
