# Supplementary material for: Pandemic-Related Impairment in the Monitoring of Patients With Hypertension and Diabetes and the Development of a Digital Solution for the Community Health Worker: Quasiexperimental and Implementation Study
Source: JMIR Med Inform. 2022 Mar 29;10(3):e35216. doi: 10.2196/35216 (PMC8966891; doi:10.2196/35216)
Supplement: Multimedia Appendix 1 [file medinform_v10i3e35216_app1.docx]

**MULTIMEDIA APPENDIX 1**

Characteristics of Experts who validated the app (N=7).

| **Characteristic** | | **Value** |
| --- | --- | --- |
| Gender, female, n (%) | | 5 (71.4) |
| Age in years, median (IQR) | | 32.0 (25.0, 43.0) |
| Education level (higher education), n (%) | | 6.0 (85,7) |
| **Time since graduation, n (%)** | |  |
|  | < 1 year | 3.0 (50) |
|  | 10-15 | 2.0 (33.3) |
|  | >15 | 1.0 (16.7) |
| **Profession, n (%)** | |  |
|  | Medical Doctor | 4.0 (57.1) |
|  | Nurse | 1.0 (14.3) |
|  | Pharmacist | 1.0 (14.3) |
|  | CHW ^a^ | 1.0 (14.3) |
| **Occupation area, n (%)** | | 409 (37.9) |
|  | PC ^b^ patient assistance | 5.0 (71.4) |
|  | PC patient assistance and health management | 2.0 (28.6) |
| **Self-reported knowledge of information technology, n (%)** | |  |
|  | Excellent | 5.0 (71.4) |
|  | Satisfactory | 2.0 (28.6) |

Statistics presented: n (%); Median (IQR)

^a^ CHW – Community health worker

^b^ PC – Primary care
