# Supplementary material for: Pandemic-Related Impairment in the Monitoring of Patients With Hypertension and Diabetes and the Development of a Digital Solution for the Community Health Worker: Quasiexperimental and Implementation Study
Source: JMIR Med Inform. 2022 Mar 29;10(3):e35216. doi: 10.2196/35216 (PMC8966891; doi:10.2196/35216)
Supplement: Multimedia Appendix 2 [file medinform_v10i3e35216_app2.docx]

**MULTIMEDIA APPENDIX 2**

Feasibility, usability and utility assessment

| Item | | Overall (n=7)  Median (IQR) | Physicians (n=4)  Median (IQR) | Nurse (n=1) | Pharmacist (n=1) | CHW (n=1) |
| --- | --- | --- | --- | --- | --- | --- |
| **Feasibility** | |  |  |  |  |  |
|  | The application can be used in the primary care setting to improve care for people with hypertension and/or DM ^a^ | 5.0 (5.0,5.0) | 5.0 (5.0,5.0) | 5.0 | 5.0 | 4.0 |
|  | It is easy to incorporate in work routine. | 4.0 (4.0,5.0) | 4.5 (4.0,5.0) | 4.0 | 4.0 | 3.0 |
|  | The app does not cause significant delays in daily routine. | 5.0 (4.0,5.0) | 5.0 (5.0,5.0) | 4.0 | 4.0 | 2.0 |
|  | Internet connection is not essential for the use of the app. | 5.0 (5.0,5.0) | 5.0 (5.0,5.0) | 5.0 | 5.0 | 2.0 |
| **Usability** | |  |  |  |  |  |
|  | My overall evaluation of the app is good. | 4.0 (4.0,5.0) | 5.0 (4.25,5.0) | 4.0 | 4.0 | 4.0 |
|  | The app is intuitive and requires no previous training to use. | 3.0 (2.0,4.0) | 3.50 (2.25,4.75) | 2.0 | 4.0 | 1.0 |
|  | The app screens are easy to understand | 4.0 (2.0,5.0) | 4.5 (4.0,5.0) | 2.0 | 4.0 | 1.0 |
|  | I was able to find the information I was looking for while using it. | 4.0 (4.0,5.0) | 5.0 (4.25,5.0) | 4.0 | 5.0 | 1.0 |
|  | The app fields are easy to complete | 5.0 (4.0,5.0) | 5.0 (5.0,5.0) | 4.0 | 5.0 | 1.0 |
|  | The app has a uniform and suitable interface | 5.0 (4.0,5.0) | 5.0 (4.25,5.0) | 5.0 | 5.0 | 1.0 |
|  | The app is stable, and no errors occur during use | 5.0 (5.0,5.0) | 5.0 (5.0,5.0) | 5.0 | 5.0 | 4.0 |
| **Utility** | |  |  |  |  |  |
|  | I believe that the app might improve the treatment of people with hypertension and DM. | 5.0 (5.0,5.0) | 5.0 (5.0,5.0) | 5.0 | 5.0 | 4.0 |
|  | Reading the recommendations of the app, the CHW ^b^ might contribute to the improvement of care for people with hypertension and DM. | 5.0 (5.0,5.0) | 5.0 (5.0,5.0) | 5.0 | 5.0 | 2.0 |
|  | The app is useful to identify people at risk for complications such as cardio or cerebrovascular events and DM complications (ketoacidosis, hyperosmolar state) | 5.0 (5.0,5.0) | 5.0 (5.0,5.0) | 5.0 | 5.0 | 4.0 |
|  | The app is useful to improve the municipality's management of medication supply for hypertension and DM | 5.0 (4.0,5.0) | 5.0 (4.25,5.0) | 5.0 | 5.0 | 3.0 |
|  | The app is useful to assist in the treatment of patients | 5.0 (5.0,5.0) | 5.0 (5.0,5.0) | 5.0 | 5.0 | 1.0 |
|  | According to my previous knowledge, I believe the recommendations generated by the app are appropriate. | 5.0 (5.0,5.0) | 5.0 (5.0,5.0) | 5.0 | 5.0 | 1.0 |

Statistics presented: Median(IQR)

^a^ DM – Diabetes mellitus

^b^ CHW – Community health worker
