# Supplementary material for: Pandemic-Related Impairment in the Monitoring of Patients With Hypertension and Diabetes and the Development of a Digital Solution for the Community Health Worker: Quasiexperimental and Implementation Study
Source: JMIR Med Inform. 2022 Mar 29;10(3):e35216. doi: 10.2196/35216 (PMC8966891; doi:10.2196/35216)
Supplement: Multimedia Appendix 3 [file medinform_v10i3e35216_app3.docx]

**MULTIMEDIA APPENDIX 3**

Characteristics of the study patients

| Characteristic | | Overall | DM ^a^ | Hypertension |
| --- | --- | --- | --- | --- |
| Number of patients, n (%) | | 1314 (100) | 245 (18.6) | 1,266 (96.4) |
| Gender, female n (%) | | 899 (68.4) | 180.0 (73.5) | 868.0  (68.6) |
| Age in years, median (IQR) | | 64.0 (54.0, 73.0) | 66.0 (55.5, 73.0) | 65.0 (54.0, 74.0) |
| **Daily use of medication in the last 7 days, n (%)** | |  |  |  |
|  | Yes | 1268.0 (96.5) | 234.0 (95.5) | 1225.0 (96.8) |
|  | No | 43.0 (3.3) | 10.0 (4.1) | 39.0 (3.1) |
|  | Do not know | 3.0 (0.2) | 1.0 (0.4) | 2.0 (0.2) |
| **Use of the correct dose of medication in the last 7 days, n (%)** | |  |  |  |
|  | Yes | 1264.0 (96.2) | 238.0 (97.1) | 1219.0 (96.3) |
|  | No | 47.0 (3.6) | 6.0 (2.4) | 45.0 (3.6) |
|  | Do not know | 3.0 (0.2) | 1.0 (0.4) | 2.0 (0.2) |
| **Reason for not using the medication correctly (among those who do not use correct dose), n (%)** | |  |  |  |
|  | Medications are over | 5.0 (10.7) | 0.0 (0.0) | 5.0 (11.1) |
|  | Unable to use medication on their own | 15.0 (31.9) | 5.0 (83.3) | 13.0 (28.9) |
|  | Medications caused side effects | 1.0 (2.1) | 0.0 (0.0) | 1.0 (2.2) |
|  | Other reasons | 26.0 (55.3) | 1.0 (16.7) | 26.0 (57.8) |
|  | Missing cases | 0.0 (0.0) | 0.0 (0.0) | 0.0 (0.0) |
| **Adequate supply of medication,**  **n (%)** | |  |  |  |
|  | Yes | 1272.0 (96.8) | 238.0 (97.1) | 1228.0 (97.0) |
|  | No | 38.0 (2.9) | 6.0 (2.4) | 35.0 (2.8) |
|  | Do not know | 4.0 (0.3) | 1.0 (0.4) | 3.0 (0.2) |
| **Those who do not have adequate supply, have an up-to-date prescription, n (%)** | |  |  |  |
|  | Yes | 24.0 (63.2) | 4.0 (66.7) | 23.0(65.7) |
|  | No | 14.0 (36.8) | 2.0 (33.3) | 12.0(34.3) |
| **SBP^b^ greater than or equal to 160 mmHg or DBc^b^ greater than or equal to 100 mmHg, n (%)** | |  |  |  |
|  | Yes | NA^d^ | NA | 220 (17.4) |
|  | No |  |  | 1045 (82.5) |
|  | Do not know |  |  | 1 (0.1) |
| **Insulin prescription, n (%)** | | |  |  |
|  | Yes | NA | 50.0 (20.4) | NA |
|  | No |  | 188.0 (76.7) |  |
|  | Do not know |  | 7.0 (2.9) |  |
| **Daily use of insulin in the last 7 days, n (%)** | |  |  |  |
|  | Yes | NA | 42.0 (84.0) | NA |
|  | No |  | 8.0 (16.0) |  |
|  | Do not know |  | 0.0 (0.0) |  |
| **Glucometer at home, n (%)** | | |  |  |
|  | Yes | NA | 60.0 (24.5) | NA |
|  | No |  | 179.0 (73.1) |  |
|  | Do not know |  | 6.0 (2.4) |  |
| **Capillary blood glucose strips at home, n (%)** | |  |  |  |
|  | Yes | NA | 54.0 (22.0) | NA |
|  | No |  | 5.0 (2.0) |  |
|  | Do not know |  | 1.0 (0.4) |  |
|  | Missing |  | 185 (75.5) |  |
| **Capillary blood glucose at any time greater than or equal to 250 mg/dl in the last 20 days** | |  |  |  |
|  | Yes | NA | 34.0 (13.9) | NA |
|  | No |  | 181.0 (73.9) |  |
|  | Do not know |  | 30.0 (12.2) |  |
| **Positive glycosuria at any time, n (%)** | |  |  |  |
|  | Yes | NA | 40.0 (16.3) | NA |
|  | No |  | 165.0 (67.4) |  |
|  | Do not know |  | 40.0 (16.3) |  |

Statistics presented: n (%); Median (IQR)

^a^ Diabetes mellitus

^b^Systolic blood pressure

^c^ Diastolic blood pressure

^d^ Not applicable

It is noteworthy that DM and hypertension coexist in 197 patients.
